# Cerebrocortical activation following unilateral labyrinthectomy in mice characterized by whole-brain clearing: implications for sensory reweighting

**DOI:** 10.1038/s41598-022-19678-4

**Published:** 2022-09-14

**Authors:** Ryota Kai, Kuniyuki Takahashi, Kazuki Tainaka, Yuriko Iwakura, Hisaaki Namba, Nae Saito, Toshikuni Sasaoka, Shun Yamaguchi, Hiroyuki Nawa, Arata Horii

**Affiliations:** 1 Department of Otolaryngology Head and Neck Surgery, Niigata University Graduate School of Medical and Dental Sciences, 1 Asahi-machi, Chuo-ku, Niigata, 951-8510 Japan; 2 Department of Molecular Neurobiology, Brain Research Institute, Niigata University, Niigata, Japan; 3 Department of System Pathology for Neurological Disorders, Brain Research Institute, Niigata University, Niigata, Japan; 4 Department of Brain Tumor Biology, Brain Research Institute, Niigata University, Niigata, Japan; 5 Department of Comparative and Experimental Medicine, Brain Research Institute, Niigata University, Niigata, Japan; 6 Department of Morphological Neuroscience, Gifu University Graduate School of Medicine, Gifu, Japan

**Keywords:** Neuroscience, Molecular medicine, Neurology

## Abstract

Posture and gait are maintained by sensory inputs from the vestibular, visual, and somatosensory systems and motor outputs. Upon vestibular damage, the visual and/or somatosensory systems functionally substitute by cortical mechanisms called “sensory reweighting”. We investigated the cerebrocortical mechanisms underlying sensory reweighting after unilateral labyrinthectomy (UL) in mice. Arc-dVenus transgenic mice, in which the gene encoding the fluorescent protein dVenus is transcribed under the control of the promoter of the immediate early gene Arc, were used in combination with whole-brain three-dimensional (3D) imaging. Performance on the rotarod was measured as a behavioral correlate of sensory reweighting. Following left UL, all mice showed the head roll-tilt until UL10, indicating the vestibular periphery damage. The rotarod performance worsened in the UL mice from UL1 to UL3, which rapidly recovered. Whole-brain 3D imaging revealed that the number of activated neurons in S1, but not in V1, in UL7 was higher than that in sham-treated mice. At UL7, medial prefrontal cortex (mPFC) and agranular insular cortex (AIC) activation was also observed. Therefore, sensory reweighting to the somatosensory system could compensate for vestibular dysfunction following UL; further, mPFC and AIC contribute to the integration of sensory and motor functions to restore balance.

## Introduction

Following unilateral damage to the vestibular inner ear, oculomotor and postural symptoms such as nystagmus and postural instability occur, but gradually recover over time in a process known as vestibular compensation^1–3^. Brain plasticity is thought to be responsible for vestibular compensation, because functional recovery occurs without regeneration of the vestibular periphery. Vestibular compensation includes changes in the patterns of floccular inhibition of the vestibular nucleus complex (VNC) and the recovery of spontaneous firing and responsiveness in ipsilesional VNC neurons^2,4,5^. In addition to vestibular compensation, appropriate postural control after vestibular damage also depends on dynamic changes in multimodal sensorimotor control systems known as “sensory reweighting”^6^. Therefore, when studying the recovery after vestibular damage, it is important to consider not only vestibular compensation, in which the cerebellum-brainstem pathways are involved, but also the cerebrocortical mechanisms of sensory reweighting.

Immediately after acute vestibular insults, postural control shifts to visual and/or somatosensory dependence, which can substitute impaired vestibular function in humans^7,8^. Functional brain imaging studies have demonstrated that cerebral glucose metabolism is significantly decreased in the multisensory vestibular cortical and subcortical areas, such as the parieto-insular vestibular cortex (PIVC), but it is increased in the visual and somatosensory cortices in the chronic stage of vestibular neuritis^9^. A similar visual substitution in response to vestibular loss has also been observed in patients with bilateral vestibular failure^10^. Studies on spaceflight in humans have shown that sensory reweighting to the visual and somatosensory systems and adaptive cortical neuroplasticity occurred as a result of changes to vestibular signaling^11,12^. Sensory reweighting of visual/somatosensory dependence following these changes could be attributed to the functional changes observed in the cerebral cortices.

While cortical changes caused by vestibular stimulation and damage have been mostly assessed in humans, animal studies relevant to sensory reweighting in the vestibular system are limited. Based on the findings in humans, we investigated whether the visual and somatosensory cortices are related to sensory reweighting in mice. In particular, we focused on the barrel cortex (S1) in the somatosensory cortex because whisker sensation is superior in rodents. Since mice are genetically easy to manipulate, they are suitable as experimental animals for neuroscience research. In this study, we employed Arc-dVenus transgenic (Tg) mice, in which activated neurons were visualized using fluorescence. While Arc is not expressed in the cerebellum^13^, which is an essential site in the cerebellum-brainstem pathway for vestibular compensation, it does not interfere with the investigation of cerebral cortex mechanisms of sensory reweighting following vestibular injuries. Clear, Unobstructed Brain/Body Imaging Cocktails and Computational Analysis (CUBIC), and the light sheet fluorescence (LSF) microscope method enable tissue clearing with chemical reagents and 3D imaging of the whole brain^14,15^. Unlike brain histology with slicing, transparent CUBIC 3D whole-brain imaging provides comprehensive monitoring of the target cell population without technical loss of detection. In the present study, to investigate the cortical correlates of sensory reweighting following vestibular damage, cortical activation following chemical unilateral labyrinthectomy (UL) using arsanilate was investigated using CUBIC/LSF microscope methods in Arc-dVenus Tg mice. Chemical UL, rather than surgical UL, was chosen because surgical UL is technically difficult to perform at the small inner ear of mice. Arsanilate was used because previous studies have reported that UL using arsanilate is a suitable model to investigate the mechanisms of vestibular compensation in mice^16^.

Regions of interest (ROIs) were set at the visual cortex (V1) and somatosensory cortex (S1), which are known to substitute for vestibular loss in humans^7–9^. The posterior granular insular cortex (pGIC), a cortical area corresponding to the human/primate PIVC, was investigated following the loss of sensory inputs from the vestibular periphery. The primary auditory cortex (A1) was also investigated, since UL can affect not only the vestibular organ but also the cochlea. In addition to these preselected areas, newly identified areas that were activated following UL were also investigated. This includes the medial prefrontal cortex (mPFC), the agranular insular cortex (AIC), and the somatosensory cortex (S1). Behavioral correlates, including head roll-tilt and rotarod performance, were also investigated following UL.

## Results

### Behavioral study

### Head roll-tilt

**Figure 1:**
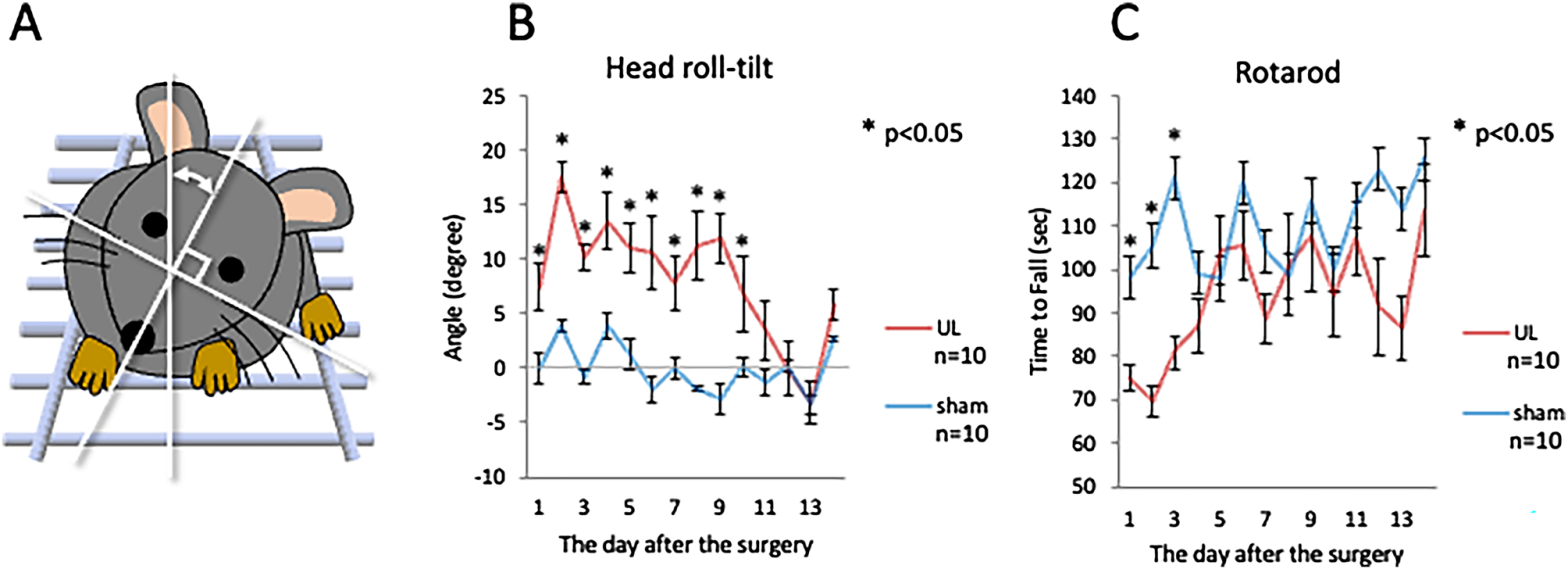
Behavioral observation. (**A**) Measurement of head roll-tilt. The angle of the sagittal plane from the vertical plane was used to evaluate vestibular imbalance. (**B**) Changes in the head roll-tilt after surgery. (**C**) Post-surgery performance in the rotarod test.

All mice that underwent left UL showed head roll-tilt towards the left side (Fig. 1A). The angle of head roll-tilt of UL mice gradually decreased, and was significantly larger than that of the sham-treated group from day 1 to day 10 following UL. The angle recovered to the same level as that exhibited by the sham-treated group by day 11 following UL (Fig. 1B).

### Rotarod test

The time to fall (TTF) in the rotarod test group was significantly shorter in the UL than in the sham-treated group during the first 3 days after UL. The TTF in the UL group rapidly recovered to the same level as that in the sham-treated group by day 4 after UL, which was faster than the recovery observed on the head roll-tilt (Fig. 1C).

### Whole-brain images

**Figure 2:**
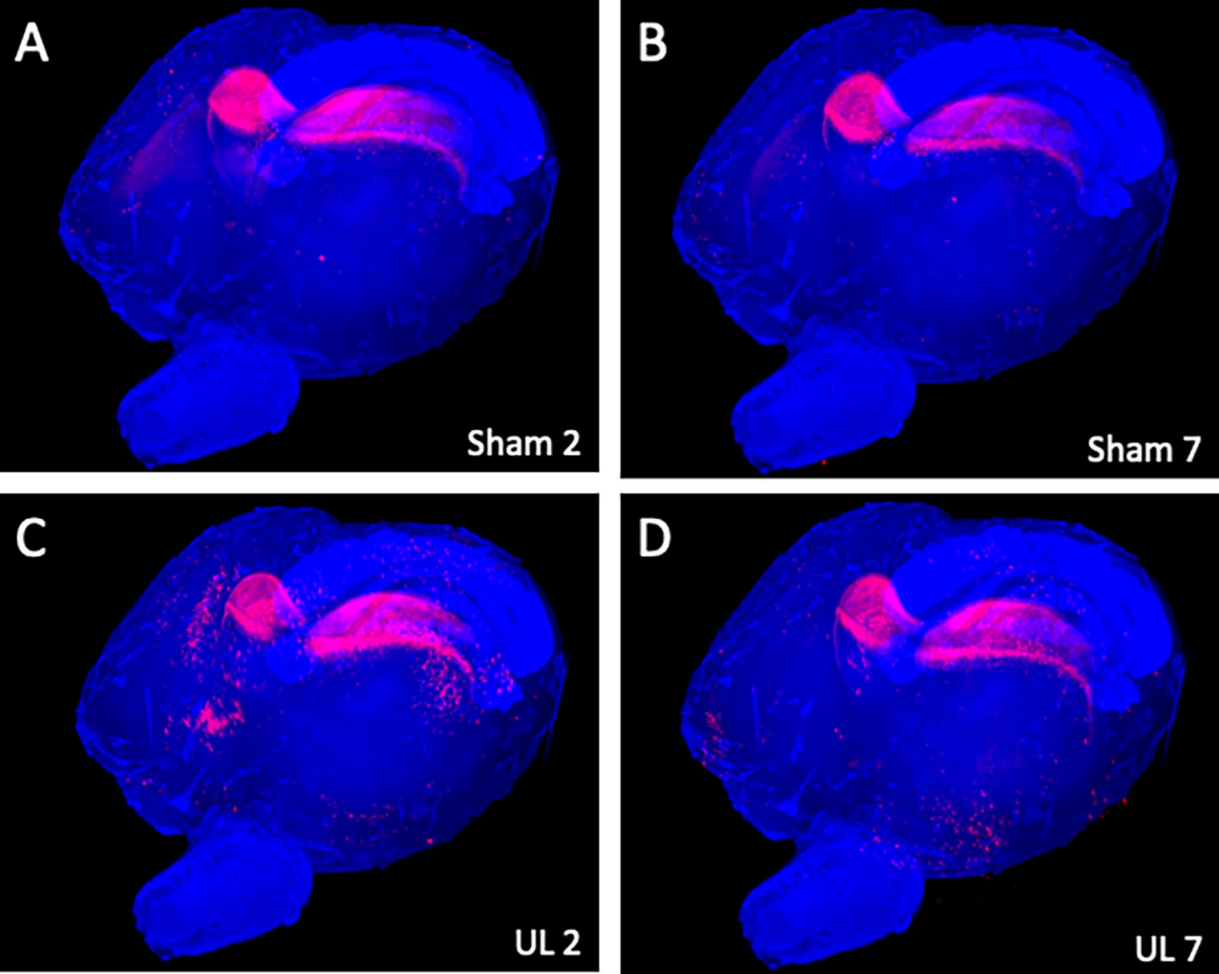
Representative whole-brain images of each group. Panoramic view of whole-brain imaging using CUBIC analysis. Red fluorescence indicates activated neurons. There were many non-specific activated neurons in the hippocampus in all images. (**A**) Images obtained 2 days after sham surgery. (**B**) Images obtained 7 days after sham surgery. (**C**) Image obtained 2 days after unilateral (left) labyrinthectomy (UL). (**D**) Image obtained 7 days after UL.

Figure 2 shows whole-brain images of sham-treated (Sham2 and Sham7) and UL individuals (UL2 and UL7) on day 2 and day 7 after surgery. Neural activation of the whole brain can be comprehensively visualized using a panoramic view, from which 3D images can also be obtained (Supplementary Movie 1). Hippocampal neurons were activated non-specifically in both groups.

### Visual cortex (V1) and somatosensory cortex (S1)

**Figure 3:**
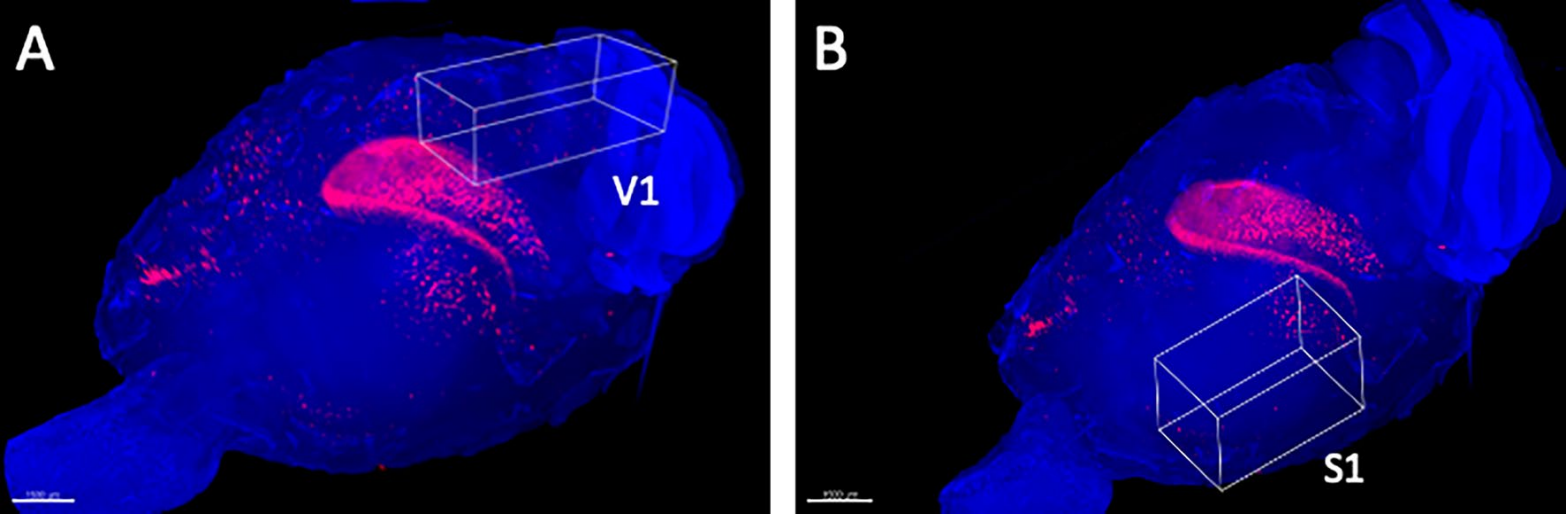
3D ROI settings in V1 and S1. (**A**) 3D ROI setting in the primary visual cortex (V1). (**B**) 3D-ROI setting in the primary somatosensory cortex (S1). Only the left hemisphere is shown, since showing both hemispheres would interfere with clarity due to image overlapping.

**Figure 4:**
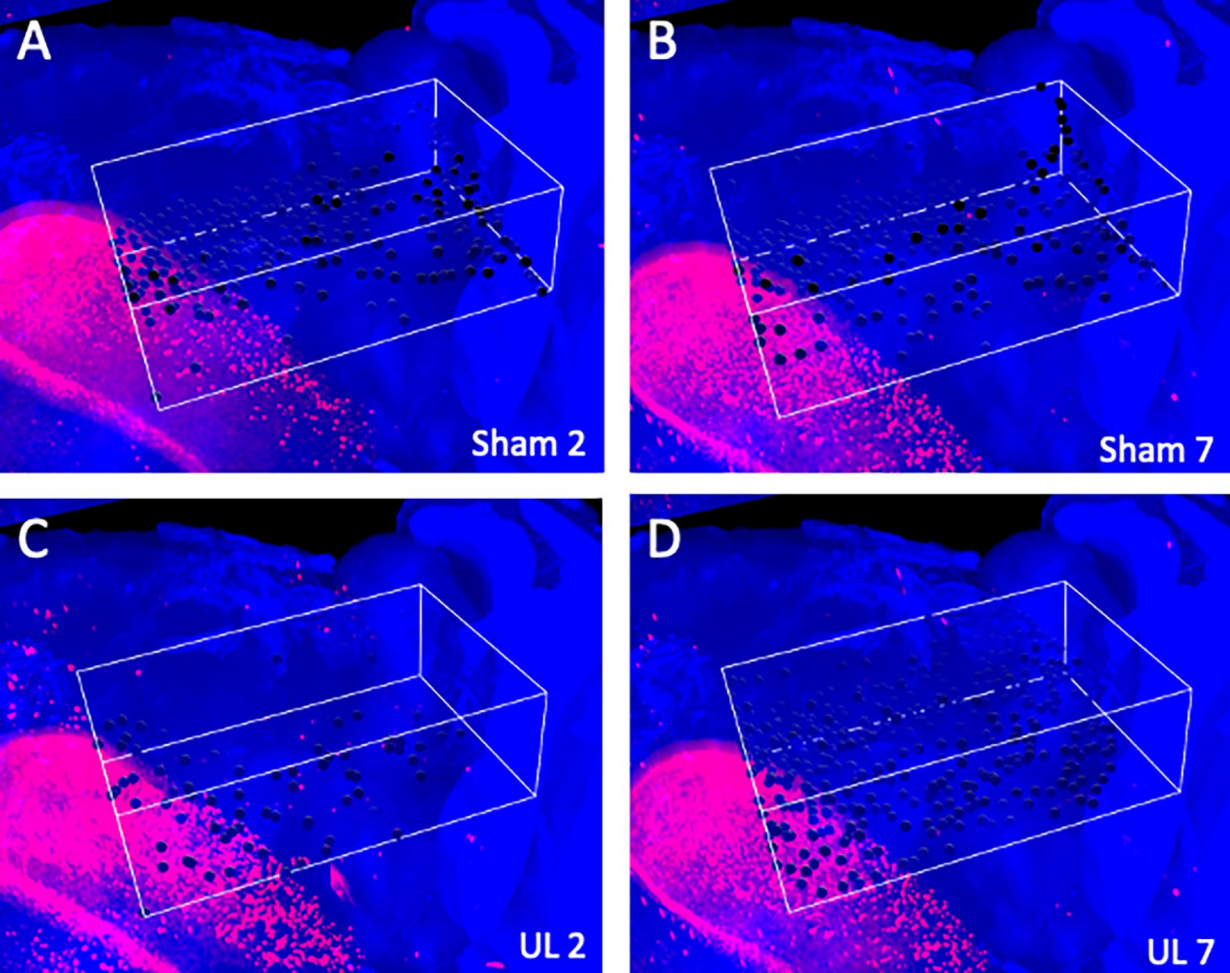
Magnified images of V1. Representative images of activated neurons in box-shaped 3D ROIs set in V1. The black dots indicate automatically-selected activated neurons inside the 3D ROIs. (**A**) Images obtained 2 days after sham surgery. (**B**) Images obtained 7 days after sham surgery. (**C**) Images obtained 2 days after unilateral (left) labyrinthectomy (UL). (**D**) Images obtained 7 days after UL.

**Figure 5:**
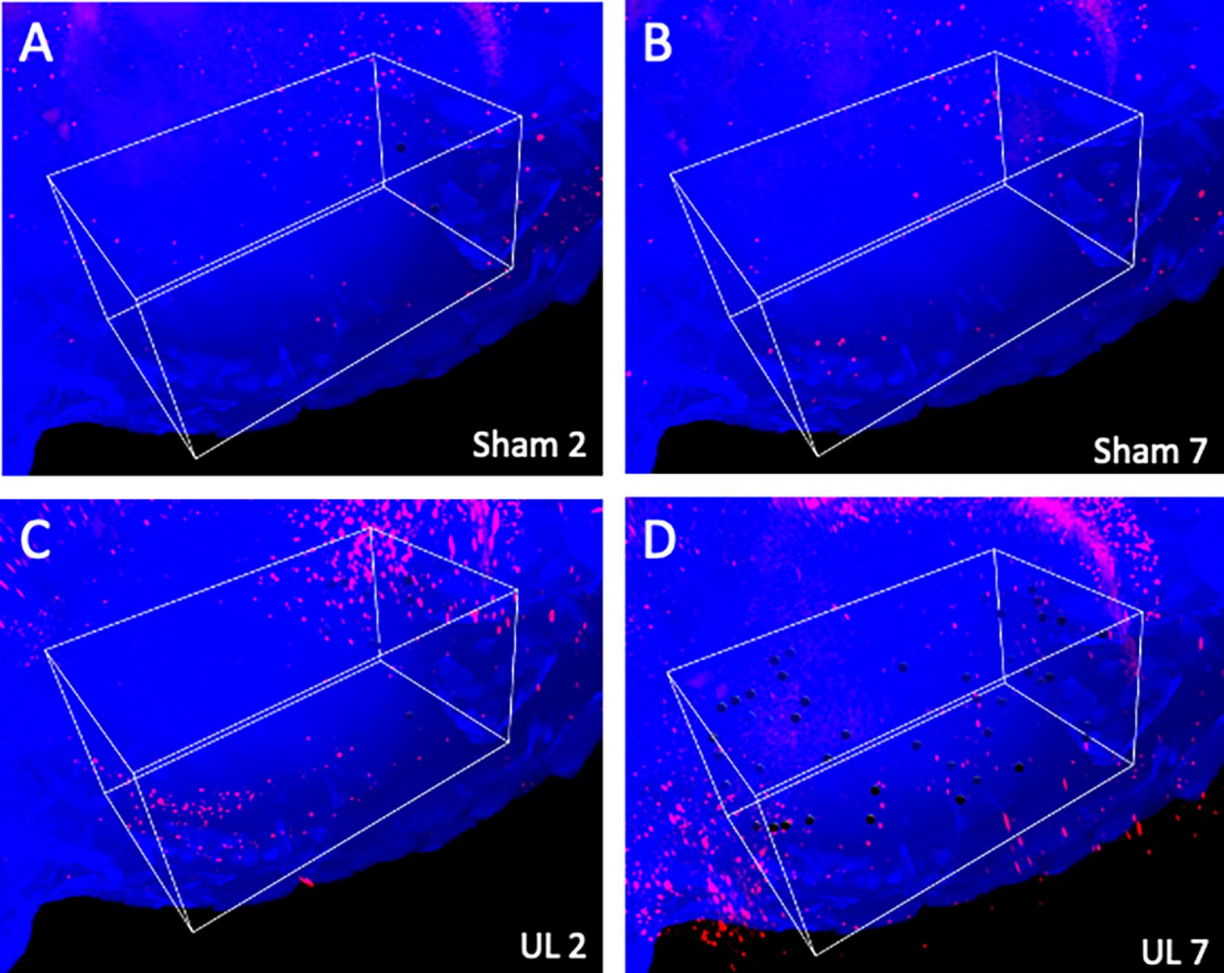
Magnified images of S1. Representative images of activated neurons in box-shaped 3D ROIs set in S1. The black dots indicated automatically-selected activated neurons inside the 3D ROIs. (**A**) Images obtained 2 days after sham surgery. (**B**) Images obtained 7 days after sham surgery. (**C**) Images obtained 2 days after unilateral (left) labyrinthectomy (UL). (**D**) Images obtained 7 days after UL.

**Figure 6:**
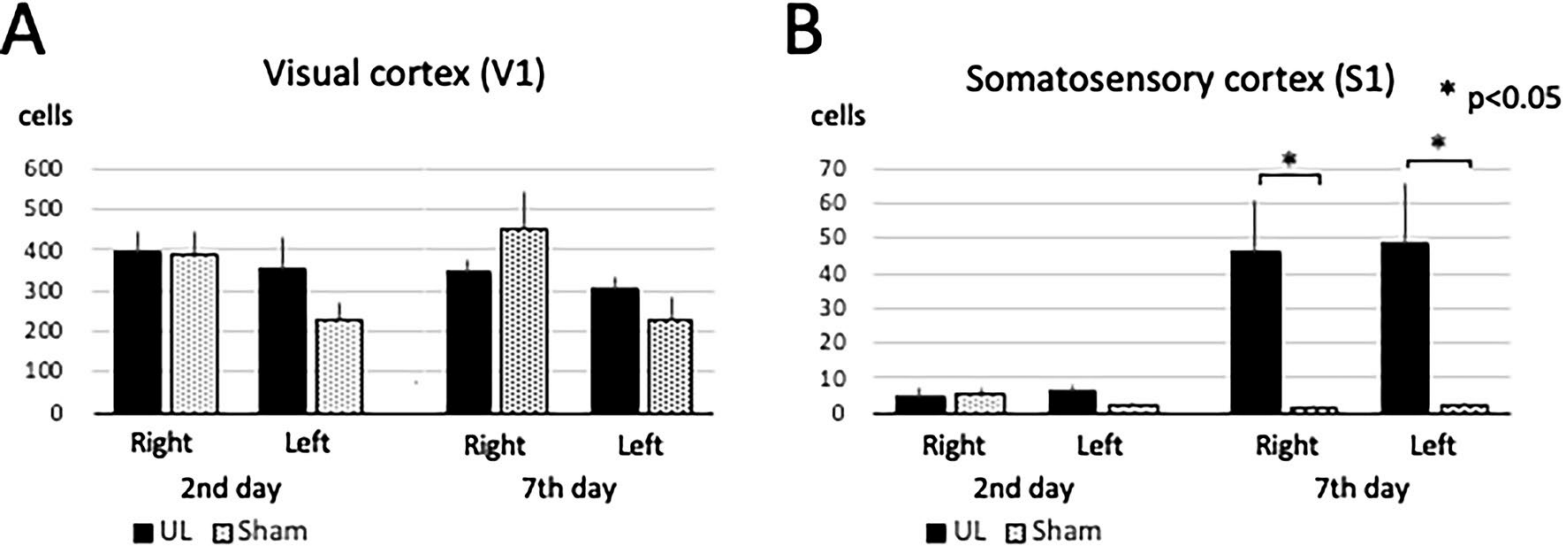
Activated neurons in V1 and S1. (**A**) Activated neurons in the visual cortex (V1). There were no significant differences in V1 between the UL and the sham-treated group. (**B**) Activated neurons in the somatosensory cortex (S1). There were significantly more activated neurons in the UL group than in the sham-treated group on day 7 after surgery. Error bars indicate the standard error of the mean.

On day 2 after surgery, there were no significant bilateral differences in the number of activated neurons in V1 and S1 between the UL and the sham-treated group. On day 7 after surgery, the number of activated neurons in the bilateral S1 was significantly higher in the UL than in the sham-treated mice, whereas no significant differences between these groups were found in V1 (Figs. 3, 4, 5, 6).

### Posterior granular insular cortex (pGIC) and auditory cortex (A1)

**Figure 7:**
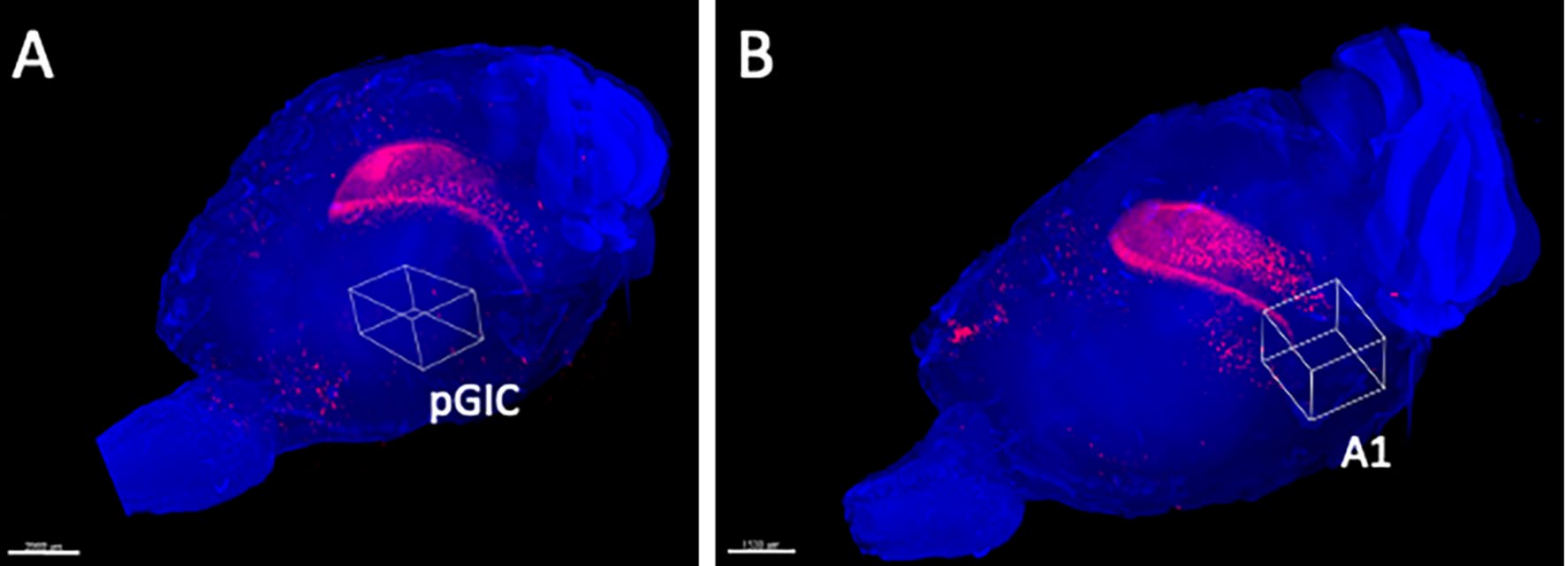
3D ROI settings in pGIC and A1. (**A**) 3D ROI setting in the posterior granular insular cortex (pGIC) as a possible vestibular cortex. (**B**) 3D ROI setting in the primary auditory cortex (A1). Only the left hemisphere is shown because showing both hemispheres would interfere with clarity due to image overlapping.

**Figure 8:**
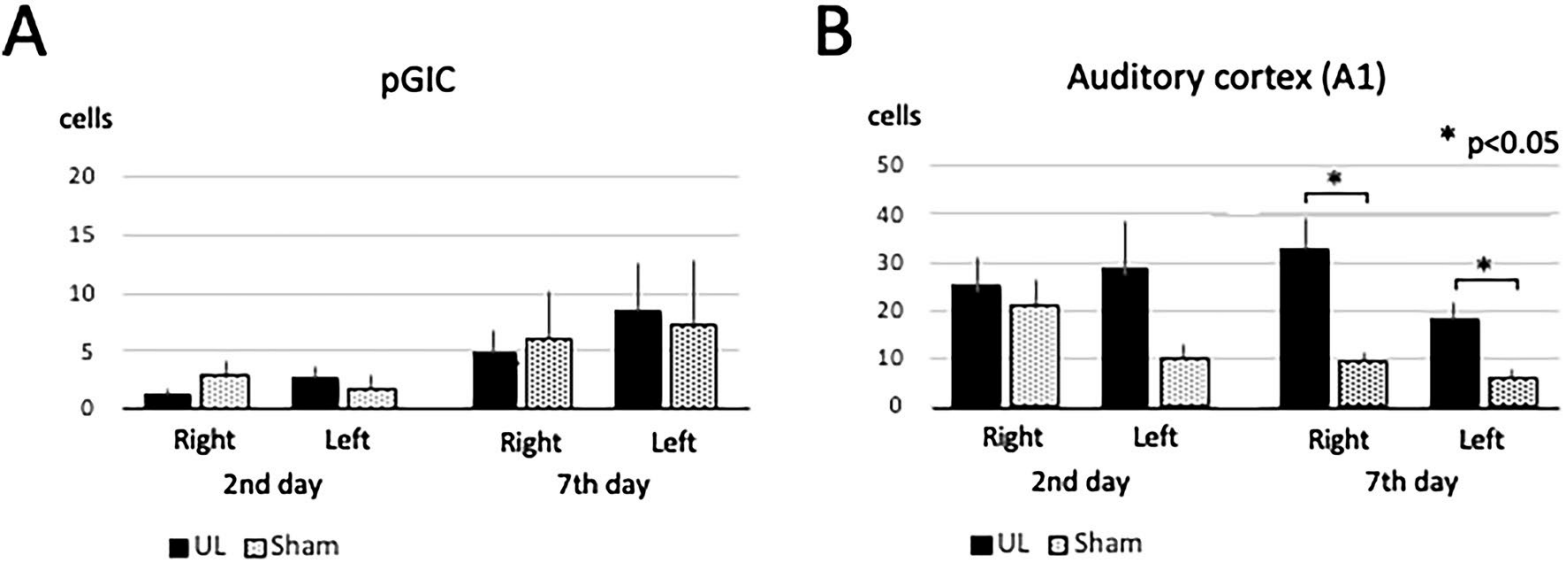
Activated neurons in pGIC and A1. (**A**) Activated neurons in the the posterior granular insular cortex (pGIC). No significant differences were observed between the UL and the sham-treated group. (**B**) Activated neurons in the auditory cortex (A1). There were no significant differences in A1 between the UL and the sham-treated group on day 2 after surgery; however, there were significantly more activated neurons in A1 of the UL group than in the sham-treated group on day 7 after surgery. Error bars indicate the standard error of the mean.

There was no significant difference in the number of activated neurons in the pGIC between the UL and the sham-treated group at both day 2 and day 7 after surgery (Figs. 7, 8A). On day 2 after surgery, there were no significant differences in the number of activated neurons in A1 between the UL and the sham-treated group. However, on day 7 after surgery, the number of activated neurons in the bilateral A1 was significantly higher in the UL group than in the sham-treated group (Figs. 7, 8B).

### Medial prefrontal cortex (mPFC) and agranular insular cortex (AIC)

**Figure 9:**
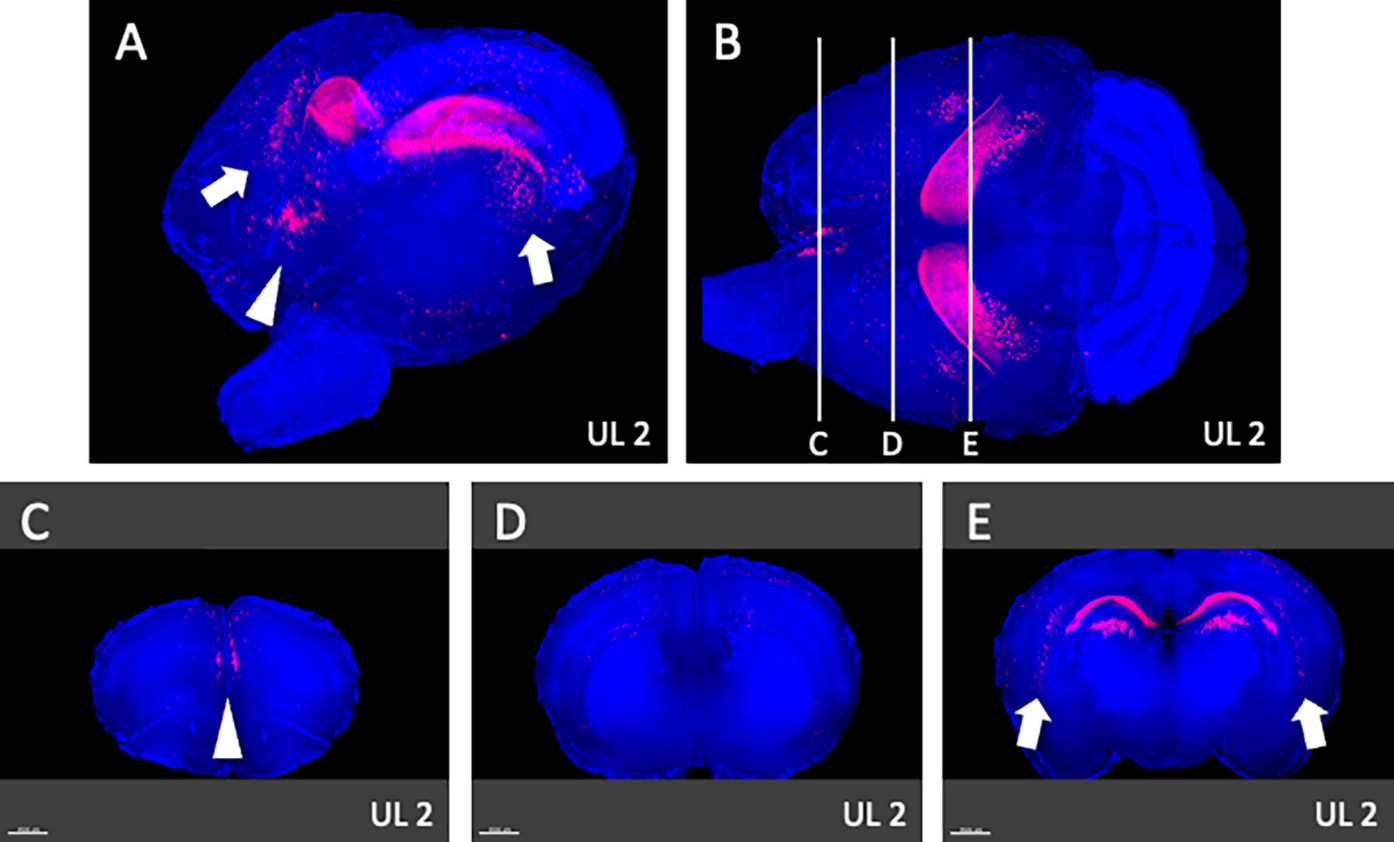
Survey on arbitrary cross sections on UL2. (**A**) Panoramic view of whole-brain imaging on day 2 after UL. Neural activity can be observed in the medial-frontal part (arrowhead) and the parietal-temporal part (arrows). (**B**) Images from the top of the brain. The vertical lines indicate the cross-sectional positions shown in panels (**C**–**E**). (**C**) Activated neurons in the medial frontal region (arrowhead, panel **A**) were located in the medial prefrontal cortex (mPFC). (**D**) A cross section of the primary somatosensory cortex (S1) region shows the absence of any activated neurons in this area. (**E**) Activated neurons in the parietal-temporal region (arrows, panel **A**) were located in the caudate putamen (arrows).

**Figure 10:**
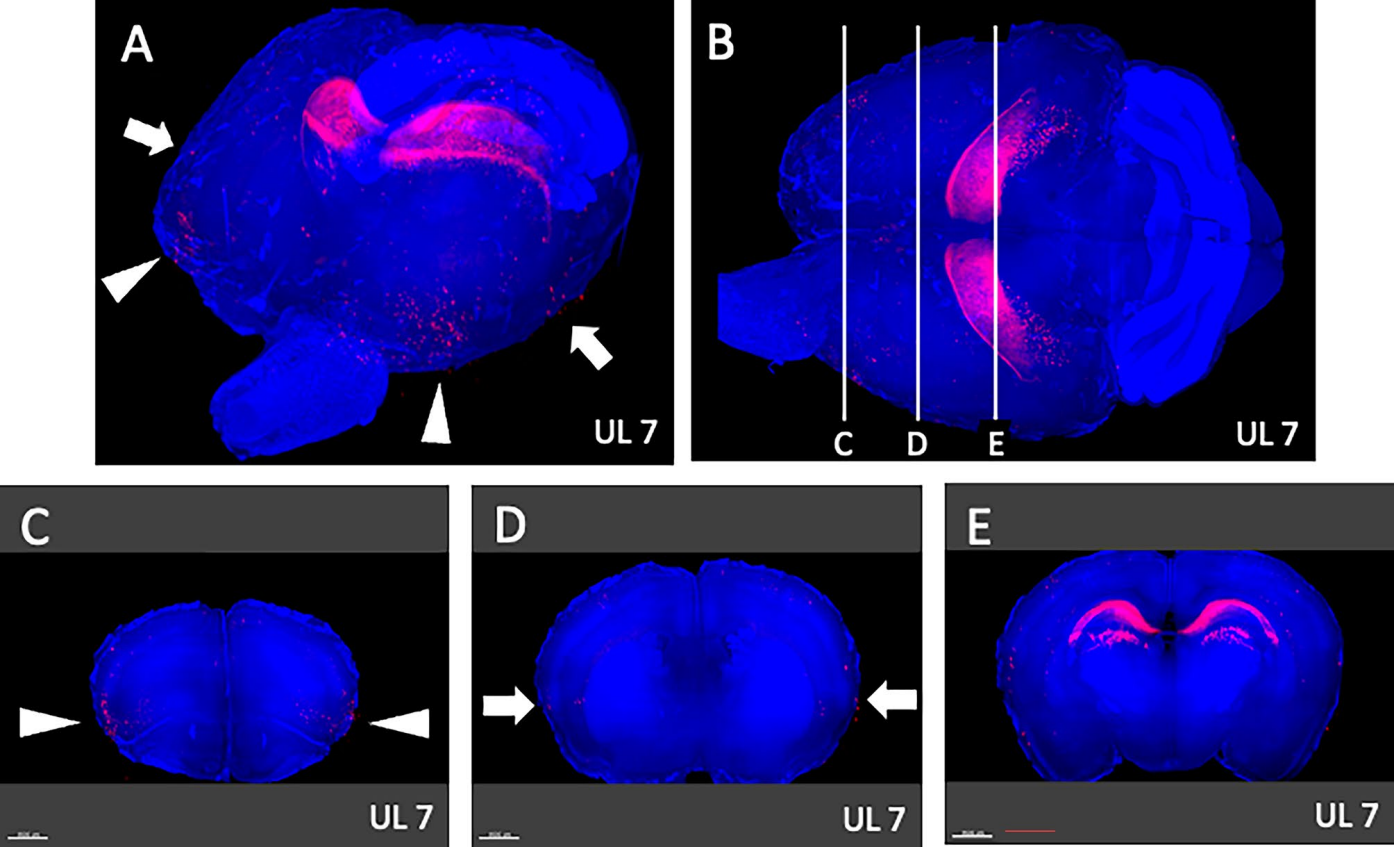
Survey on arbitrary cross sections on UL7. (**A**) Panoramic view of whole-brain imaging on day 7 after UL. Neural activity can be observed in the anterior-temporal (arrowheads) and temporal (arrows) regions. (**B**) Images from the top of the brain. The vertical lines indicate the cross-sectional positions in panels (**C**–**E**). (**C**) The activated neurons in the anterior temporal region (arrowheads, panel **A**) are located in the agranular insular cortex (AIC) (arrowheads). (**D**) Activated neurons in the temporal region (arrows, panel **A**) were located in the primary somatosensory cortex (S1). (**E**) Activated neurons observed in the caudate putamen on day 2 were decreased on day 7 after UL. Activated neurons in the caudate putamen were not observed.

**Figure 11:**
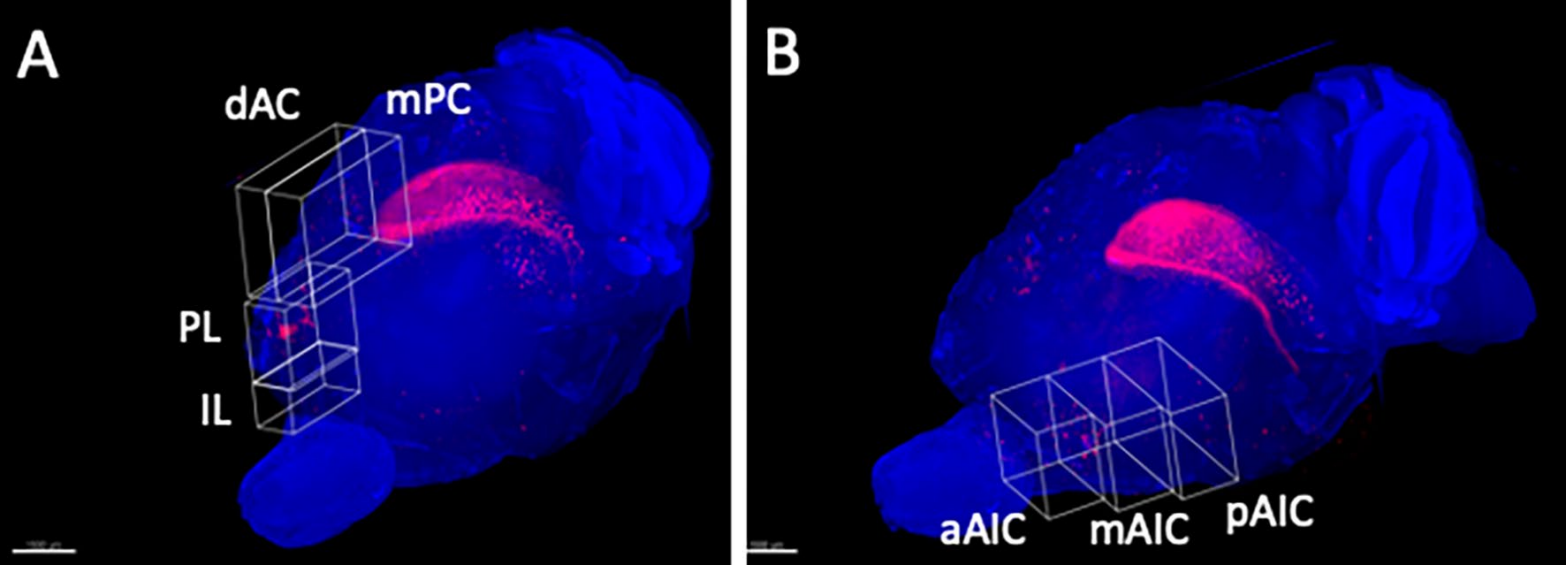
3D ROI settings in mPFC and AIC. (**A**) 3D ROI setting in the medial prefrontal cortex (mPFC). The mPFC in rodents is anatomically and functionally divided into four subdivisions from dorsal to ventral: the medial precentral cortex (mPC), dorsal anterior cingulate cortex (dAC), prelimbic cortex (PL), and inflalimbic cortex (IL). 3D ROIs were set in these four regions. (**B**) 3D ROI setting in the agranular insular cortex (AIC). The AIC is divided into three subregions, from rostral to caudal (anterior [aAIC], middle [mAIC], and posterior [pAIC]). 3D ROIs were set in these three regions. Only the left hemisphere is shown because showing both hemispheres would interfere with clarity due to image overlapping.

**Figure 12:**
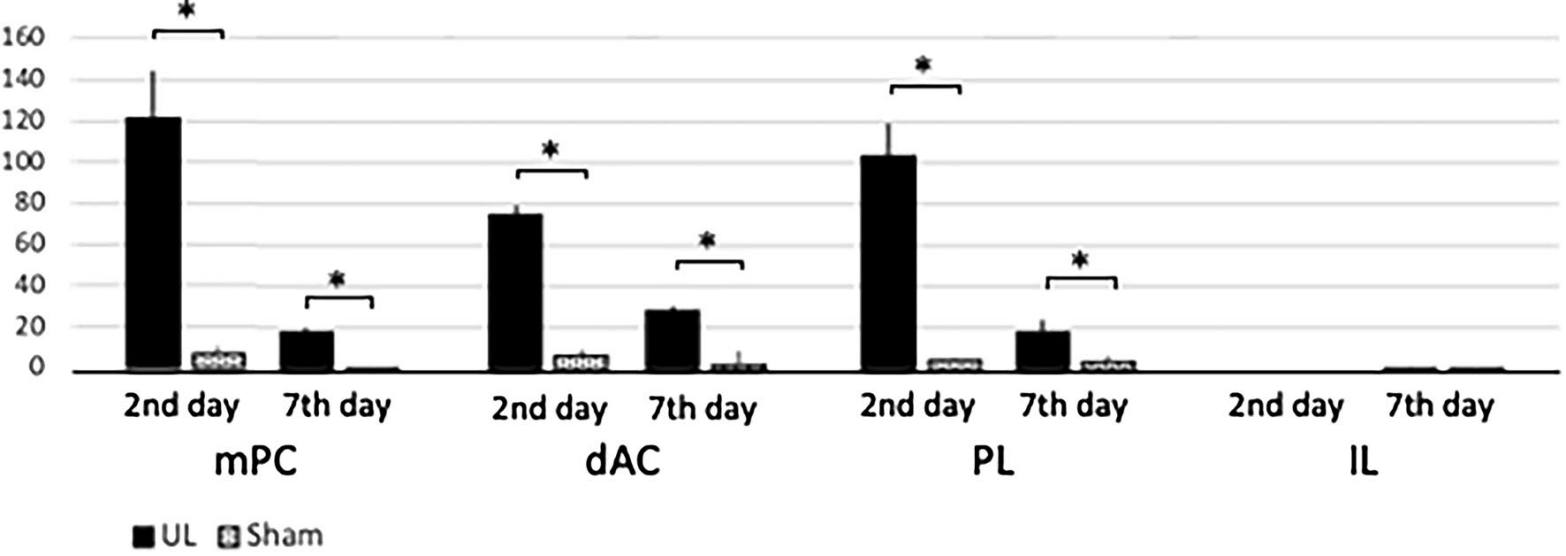
Activated neurons in mPFC. There were significantly more activated neurons in the mPC, dAC, and PL on both day 2 and day 7 after UL, but not in the IL. Error bars indicate the standard error of the mean.

**Figure 13:**
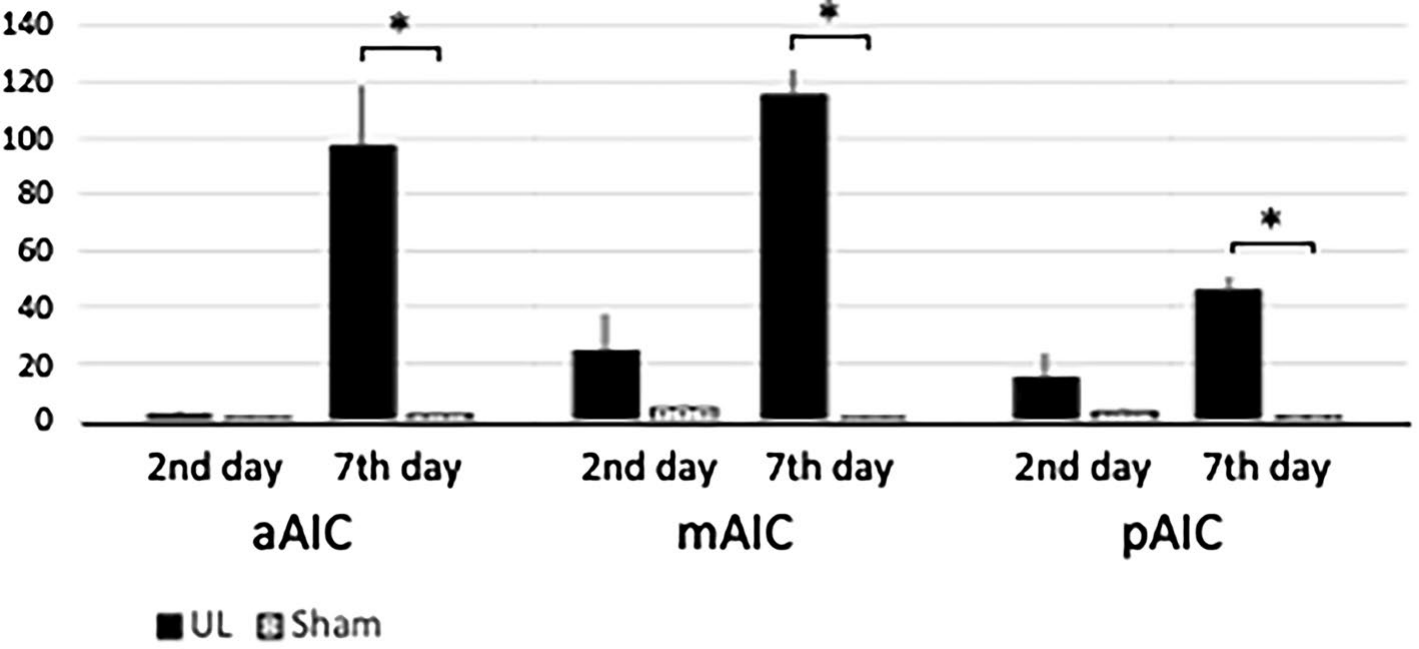
Activated neurons in AIC. There were no significant differences between the UL and the sham-treated group for any of the AIC subregions on day 2 after UL. However, there were significantly more activated neurons in all of the subregions on day 7 after UL. Error bars indicate the standard error of the mean.

Whole-brain analysis under comprehensive and panoramic views demonstrated that activated neurons were found bilaterally in the medial-frontal cortex (Fig. 9A; arrowhead) and the parieto-temporal region (Fig. 9A; arrows) on UL2. A survey of arbitrary cross-sections (Fig. 9B–E) revealed that the activated neurons were located in the mPFC (Fig. 9C) and the caudate putamen (Fig. 9E). On UL7, activated neurons could also be found bilaterally in the anterior-temporal region (Fig. 10A; arrowheads) and the temporal region (Fig. 10A; arrows). A survey of arbitrary cross sections from UL7 (Fig. 10B–E) revealed that the activated neurons were located in the agranular insular cortex (AIC) (Fig. 10C) and in S1 (Fig. 10D), which is a target area for sensory reweighting. There were a few activated neurons in these areas in individuals Sham2 and Sham7 (data not shown). Regarding the cell count of the four subregions in the mPFC (Figs. 11, 12), the number of activated neurons was significantly higher in the UL group than in the sham-treated group on day 2 after surgery in the medial precentral cortex (mPC), dorsal anterior cingulate cortex (dAC), and prelimbic cortex (PL). This number gradually decreased, but it was still significantly higher in the UL group on day 7 (Fig. 12). A few activated neurons were observed in the infralimbic cortex (IL). In the cell count of the three subregions in AIC (Figs. 11, 13), there were no significant differences between the groups on day 2, but the number of activated neurons in all the subregions was higher in mice from the UL group on day 7 compared to sham-treated mice.

## Discussion

All the mice that underwent chemical UL showed ipsilesional head roll-tilt and impaired rotarod performance. Although the present study did not assess direct vestibular damages, these behavioral results were similar to those from a previous study using the same concentration of arsanilate in which peripheral vestibular organ damages were confirmed histologically^16^. The head roll-tilt peaked on day 2 and lasted up to 10 days after the UL (Fig. 1B). Ito et al. reported that the head roll-tilt peaked on day 2 and recovered by day 4 after UL in mice that had been administered arsanilate using a post-auricular approach. The effects of intratympanic injection of arsanilate were persistent in this study compared to the administration of the same arsanilate dose using a post-auricular approach^16^. This difference may therefore be explained by the different routes of administration. Intratympanic injection without skin incision may cause the arsanilate to be retained for longer in the tympanic cavity, resulting in stronger effects on the vestibular periphery. The rotarod test has been widely used to investigate vestibular imbalance after labyrinthectomy^17^ and motor deficits due to brain injury^18,19^. In our results, rotarod performance was disrupted from day 1 to day 3, and had recovered by day 4 after UL (Fig. 1C). The recovery observed on the rotarod performance was much faster than that for the head roll-tilt. Since the head roll-tilt is mainly controlled by otolith inputs, the recovery of the head roll-tilt may be directly proportional to vestibular compensation^16^. On the other hand, the rotarod performance seems to be related not only to vestibular function, but also to motor control and coordination by other sensory modalities, including sensory reweighting. Taken together, these results suggest that vestibular compensation had not yet been completed between days 2 to 10 after UL, and that sensory reweighting occurred on day 4 after UL. Our investigation of neural activation in the cerebral cortices revealed no significant differences in V1 and S1 between the UL and the sham-treated group on day 2, but significantly higher neural activity at S1 in the UL group on day 7. The results of the neural activity at S1 in UL animals are in agreement with the results of the rotarod behavioral tests.

Our study showed that on day 7 after UL, the number of activated neurons in both the right and left somatosensory cortices (S1) was significantly higher in UL than in sham-treated animals, while no significant differences were found on day 2 (Fig. 6B). Neural activation in S1 was not only noticed when the region was pre-selected as a potential area for sensory reweighting (Figs. 3B, 5), but also when a series of arbitrary cross-sections where scanned for signs of neural activation after UL (Fig. 10D). According to the rotarod results, sensory reweighting started not earlier, but later than day 4 after UL, and it was already present on day 7 after UL (Fig. 1C). Taking these results together, activation of the somatosensory cortex (S1) (Figs. 5, 6B) may play a role in sensory reweighting after UL in mice. In contrast, there were no significant differences in the number of activated neurons in either the right or left visual cortex (V1) between the UL and sham-treated groups on day 2 or on day 7 after UL (Figs. 4, 6A), demonstrating that the visual cortex (V1) was not activated after UL. This result suggests that sensory reweighting after vestibular loss in mice may not depend on visual input. According to motor function tests, including rotarod tests performed in normal and visually impaired mice with and without somatosensory inputs from the whiskers, the somatosensory inputs from whiskers are more essential for maintaining motor function in mice^20^. These results strongly support our findings that somatosensory (whisker) inputs play a key role in sensory reweighting after UL in mice, unlike the mechanism of sensory reweighting in humans. Although there is no evidence of direct neural connections from the peripheral vestibule or vestibular nuclei to S1 and V1, multisensory cortical areas in the mouse homologous to areas 2v, 3a, and PIVC in monkeys would convey vestibular, somatosensory, and visual information to S1 and V1^21^.

It has been reported for rodents that the parieto-temporal (PT) region receives most of the projections from the vestibular nuclei^22^. However, there have been no reports on the vestibular cortex in mice. In this study, we assumed that the vestibular cortex in mice might be located in the pGIC, which is anatomically homologous to the vestibular cortex of other species (PIVC, ASSS, and PT). We set a 3D ROI for the pGIC as a potential vestibular cortex in mice. However, activated neurons were not found in the pGIC after the UL (Fig. 9A). It is suggested that the mouse vestibular cortex could not be identified in the pGIC using a vestibular deafferentation model, and that alternative research models, such as vestibular stimulation models, may be required to identify the vestibular cortex in mice.

Our study showed a significant increase in the number of activated neurons in the auditory cortex (A1) in mice on day 7 after UL, but not on day 2 (Fig. 9B). Because both UL and sham-treated animals were kept in the same conditions in terms of auditory input 2 days before decapitation, this difference was not due to reactivation by cochlear inputs from the healthy side. It may be considered that this is not the result of sensory reweighting but of disinhibition in A1, induced by the loss of peripheral sensory inputs due to cochlear damage by the UL.

In the survey on arbitrary cross sections from whole-brain imaging, the activated neurons were found in the mPFC on day 2 and day 7 after UL (Fig. 12). In rodents, the mPFC is divided into four subdivisions from dorsal to ventral: mPC, dAC, PL, and IL^23–25^ (Fig. 11A). The mPC and dAC are known to be linked to various motor behaviors, while PL and IL are associated with diverse emotional, cognitive, and mnemonic processes^23,26^. Stimulation of the mPC and dAC has been reported to induce movement of the eye, vibrissa, head, and hind limbs^27–30^. Therefore, the mPC and dAC in rodents are homologous to the frontal eye field, which acts as a supplementary motor and premotor cortex in primates^31–33^. The mPC overlaps with the vibrissa motor cortex (vM1) and receives numerous cortical inputs from the visual, somatosensory, auditory, parietal, retrosplenial, and orbital areas^34–37^. The mPC also projects to the superior colliculus^38^ and subcortical nuclei, involving oculomotor control^39,40^. Therefore, it is thought that the mPC plays a role in receiving multiple sensory inputs, in processing and in transforming them into motor functions^41^. In contrast to the motor-associated functions of the mPC and dAC, PL and IL are linked to the limbic system^25,31,42^ and profoundly influence visceral and autonomic activities, such as gastrointestinal motility and blood pressure control^43,44^. In our study, activated neurons were found in the mPC, dAC, and PL of the mPFC subdivision after UL (Fig. 12). Based on these neural connectivity and functions of the mPFC, the activation of the mPC and dAC at the early stage after UL may be related to changes in oculomotor control and integrative motor activity. PL activation may also be related to visceral and autonomic changes after UL. The basal ganglia (including the caudate putamen) control muscle tonus for posture, locomotion, and cognition through the basal ganglia-cortical and the basal ganglia-brainstem loops^45,46^. In our study, the activated neurons in the caudate putamen were observed in UL2 but decreased in UL7 (Figs. 9E, 10E). The results may be driven by the activation-deactivation of direct motor and postural control systems rather than sensory reweighting mechanisms.

The insular cortex (IC) comprises three different subareas, which differ in their cytoarchitecture of layer 4: the agranular (AIC), dysgranular (DIC), and granular (GIC) cortices (from ventral to dorsal). In our results, activated neurons were found in the AIC on day 7, but not on day 2 after UL. The IC has been suggested to function as a hub linking large-scale brain systems. It receives multisensory inputs from the outside environment, such as auditory, somatosensory, visual, olfactory, and gustatory information, as well as from inside the body, such as blood pressure, heartbeat, oxygenation, and input from the digestive system^47^. Gehrlach et al. divided the mouse IC into three equal subregions across its rostrocaudal extent and reported their connectivity to other cortical and subcortical regions. The middle and posterior subregions show similar connectivity with many sensory cortices and the amygdala. In contrast, the anterior subregion shows more connections to the motor cortex, prefrontal cortex, striatum, and sensory cortex^48^. In our results, activated neurons were found in all subregions (middle, posterior, and anterior) of the AIC on day 7 after UL. Neuronal activation of the somatosensory cortex (S1) (Figs. 5, 6B) may substitute for the loss of vestibular inputs following UL (that is, in sensory reweighting) leading to a recovery in the rotarod performance (Fig. 1C). Activation of the AIC (Fig. 13) may also play a role in sensory reweighting by integrating somatosensory and motor functions.

It has been reported that visual and somatosensory information are integrated at the early levels of the central nervous system such as the vestibular nuclei^49–52^, suggesting that sensory reweighting might occur at the brainstem level. Meanwhile, studies in humans have demonstrated changes of neural activities in the visual, somatosensory, and insular cortex after vestibular damage^9^, which is in partial agreement with our results: Only the somatosensory cortex, but not the visual cortex, was activated after UL, probably because rodents depend more on the sensory input from the whiskers than on visual inputs. Since how higher cortices such as V1, S1, mPFC, and IC influence the vestibular system is unknown, further research is needed.

## Materials and methods

This study was carried out in compliance with the ARRIVE guidelines, and the protocols were approved by the Institutional Animal Care and Use Committee of Niigata University (IACUC; approval codes #SA00673 and #SD00787). All experiments were performed in accordance with the guidelines and regulations provided by IACUC.

### Animals

Twenty C57BL/6J (CLEA Japan, Tokyo, Japan) and 16 Arc-dVenus male mice^53^, 8–10 weeks of age at the time of the experiments, were used for behavioral studies and whole-brain imaging studies, respectively. Because behavioral measurement could have affected the neuronal activities and CUBIC data, naive mice were used in behavioral studies. Arc-dVenus mice are transgenic heterozygotes mice that express fluorescent destabilized Venus protein under the control of the Arc immediate-early gene. The mice were housed in plastic cages with a 12-h light/dark cycle and under temperature-controlled conditions. Food and water were provided ad libitum.

### Procedure of chemical unilateral labyrinthectomy

All surgical procedures were performed under deep anesthesia with pentobarbital (65 mg/kg, i.p.). Unilateral chemical labyrinthectomy was performed by transtympanic injection of 50 μL of sodium arsanilate (0.4 M; Tokyo Chemical Industry, Tokyo, Japan) dissolved in 0.9% saline^16^ into the left tympanic cavity. Animals belonging to the sham-treated group received injections of the same volume of 0.9% saline solution.

### Behavioral studies

Twenty C57BL/6J naïve mice (10 in each group, UL and sham-treated) were used for behavioral observation. All of the mice that received intratympanic arsanilate showed behavioral abnormalities such as spontaneous nystagmus (SN) directed to the contralesional side and head roll-tilt directed to the ipsilesional side, indicating that intratympanic injection of arsanilate induced damage to the ipsilateral vestibular periphery. We did not use SN as a behavioral correlate of recovery after UL because of technical difficulties in the quantitative measurements of SN. To measure behavioral changes after left-side UL, the experimental animals were subjected to the head roll-tilt and rotarod tests. These two tests were used to estimate vestibular compensation and sensory reweighting, respectively, occurring in response to UL.

### Head roll-tilt

Head roll-tilt, defined as the angle of the sagittal plane from the vertical plane, has been previously used to evaluate vestibular imbalance^16^. We measured the head roll-tilt on photographs once a day and used it as an indicator of vestibular imbalance following UL (Fig. 1A).

### Rotarod test

The rotarod test has been widely applied to monitor motor performance in naïve mice^54,55^, in addition to changes in balance after labyrinthectomy^17^ and brain injury^18,19^. Mice were placed on the rod at a slow rotational speed (4 rpm). The speed was then gradually increased to 40 rpm over 120 s. Each session lasted 180 s. The session ceased if the mouse fell from the rod, at which point the time to fall (TTF) was recorded. The experiment consisted of three trials per day and the best performance was recorded. These measurements were performed during 14 consecutive days following UL.

### Whole-brain imaging study

Sixteen Arc-dVenus mice were used for 3D visualization using CUBIC analysis. To minimize the effects on other sensory modalities, including external visual and auditory inputs, all mice used for imaging studies were placed in a light- and sound-deprived room for two days before decapitation. Animals were decapitated on days 2 or 7 after UL or sham surgery (4 groups of 4 mice each).

### Tissue clearing

Arc-dVenus mice were anesthetized with a mixture of medetomidine hydrochloride (0.75 mg/kg body weight [BW]), midazolam (4 mg/kg BW), and butorphanol tartrate (5 mg/kg BW) on day 2 or day 7 after UL. The mice were then perfused with phosphate buffered saline (PBS pH 7.4) and 4% paraformaldehyde in PBS through the left ventricle. The brains were removed, post-fixed overnight in 4% paraformaldehyde in PBS at 4 °C and stored in PBS. The brains were rendered transparent for imaging according to the updated CUBIC method^14,56^. For 4–5 days, the brains were immersed in CUBIC-L [10/10 wt% chemical cocktail of N-butyldiethanolamine (B0725, Tokyo Chemical Industry) and Triton X-100 (12,967–45, Nacalai Tesque, Kyoto, Japan)] and shaken (80–100 rpm) at 37 °C. The brains were then washed three times with PBS at room temperature for 30 min and stained for 3 days at room temperature with RedDot™2 1:100 (#40061, Biotium, Fremont, CA, USA) in PBS containing 0.5 M NaCl. After staining, the brains were washed in PBS and shaken gently in 1:1 diluted CUBIC-R [45/30 wt% chemical cocktail of 45 wt% antipyrine (D1876, Tokyo Chemical Industry) and nicotinamide (N0078, Tokyo Chemical Industry), pH 10 adjusted by N-butyldiethanolamine] at room temperature for 6 h, and then shaken gently at room temperature overnight. Subsequently, the brains were immersed in fresh CUBIC-R and gently shaken at room temperature for at least 6 h.

### Whole-brain imaging

Fluorescent images were acquired using custom-built LSF microscopes (MVX10-LS, Olympus, Tokyo, Japan) and a 0.63 × objective lens (numerical aperture = 0.15, working distance = 87 mm) with 1–1.6 × digital zoom. The LSF microscope was equipped with lasers emitting light at 488 and 637 nm. The objective lens accompanied the stage movement in the axial direction to avoid defocusing. During image acquisition, the refractive index (RI)-matched samples were immersed in a mixture (RI = 1.525) of silicon oil HIVAC-F4 (RI = 1.555, Shin-Etsu Chemical Co., Tokyo, Japan) and mineral oil (RI = 1.467, M8410, Sigma Aldrich, St. Louis, MO, USA). Images were collected by scanning the samples in the z-direction at a step size of 10 μm. Fluorescent signals were visualized using a laser of an appropriate wavelength with a sequential shift in the light sheet focal positions. The thinnest focal point of the LSF microscope was horizontally scanned six times per plane to reduce defocusing from the beam’s Gaussian shape. Scanning was performed on either side of the illuminated arm. Images of identical horizontal positions (dVenus, bandpass emission filter: 495–540 nm; autofluorescence at 488 nm, bandpass emission filter: 600–690 nm; and RedDotTM2 at 637 nm, bandpass emission filter: 660–750 nm) were merged using customized image software (MVX10-MG-SW version 1.1.3, Olympus). To remove autofluorescence signals in the finalized dVenus images, autofluorescence images were subtracted from the dVenus images using Fiji software. Furthermore, considering that the brains swell in response to the clearing process and that the size of the swollen brain differed across the experimental groups, the brain size was calculated and normalized using the ratio of the size. Acquired brain images were overlapped using an automatic transformation algorithm^56^ for quantitative comparison. Reconstitution and analysis of volume-rendered images were conducted using the 3D visualization and image processing software Imaris (version 8.1.2, Bitplane, Zürich, Switzerland). The dVenus signal was overlaid with the RedDot™2 signal, and the cortical layers were identified by the arrangement of neuronal nuclei. A whole-brain image for each experimental group was thus created, as shown in Fig. 2.

### 3D ROIs of targeted cortical areas and cell count

ROIs were set at the bilateral visual cortex (V1) and somatosensory cortex (S1), which are known to substitute for vestibular loss in humans^7–9^. The S1 area of the mice is mainly located in the barrel cortex^57^. The PIVC has been proposed as a possible “vestibular cortex” in humans^58–62^ and monkeys^63–67^. The anterior suprasylvian sulcus (ASSS) is analogous to the PIVC in cats^68–70^. Since both cortices are located in the posterior part of the insular cortex and are surrounded by the somatosensory and auditory cortices, the pGIC was set as a possible cortical area corresponding to the human/primate PIVC and cat ASSS. Since UL could influence not only the vestibular organ but also the cochlea, the 3D ROIs were set on the pGIC as a possible “vestibular cortex” in mice, and the primary auditory cortex (A1) to investigate the effects of loss of sensory inputs from the inner ear. In addition, potentially significant areas where the number of activated neurons could change following UL were surveyed by arbitrary cross sections in Imaris. Box-shaped 3D ROIs were set up on these target areas for sensory reweighting (V1 and S1) (Fig. 3), sensory deprivation (pGIC and A1) (Fig. 6), and newly identified cortical areas (mPFC and AIC) (Fig. 7) according to the mouse brain atlas^57^. The number of activated neurons in the 3D ROIs was automatically counted after the signal threshold was set, and compared between the UL and sham-treated groups. The mPFC and the agranular insular cortex (AIC) were divided into subregions. Because the mPFC in rodents is anatomically and functionally divided into four subregions, namely, the medial precentral cortex (mPC), dorsal anterior cingulate cortex (dAC), prelimbic cortex (PL), and infralimbic cortex (IL), from dorsal to ventral^23–25^, the cell count in the mPFC was performed separately for these four subregions. Since the AIC is divided into three subregions from rostral to caudal (anterior [aAIC], middle [mAIC], and posterior [pAIC]) based on their connectivity with other cortical and subcortical regions^48^, the cell count in the AIC was performed separately for these three subregions.

### Statistical analysis

The results for the head roll-tilt and rotarod tests were analyzed with repeated measures two-way analysis of variance (ANOVA) followed by post-hoc Tukey`s tests to identify the postoperative days at which there were significant difference between the UL and sham-treated subjects. Differences in the total cell count were analyzed by the Mann–Whitney test using SPSS Statistics software (version 26.0 , IBM, Armonk, NY, USA). Statistical significance was set at *P* < 0.05.

### Ethics approval

All experiments were approved by the ethics committee of animal experiments from Niigata University and were carried out in accordance with the approved guidelines.

## Supplementary Information

Supplementary Video 1.

Supplementary Video 2.

Supplementary Legend.

## Data Availability

The datasets generated during and/or analyzed during the current study are available from the corresponding author on reasonable request.
