# Supplementary material for: Cerebrocortical activation following unilateral labyrinthectomy in mice characterized by whole-brain clearing: implications for sensory reweighting
Source: Sci Rep. 2022 Sep 14;12:15424. doi: 10.1038/s41598-022-19678-4 (PMC9474865; doi:10.1038/s41598-022-19678-4)
Supplement: Supplementary file 3 — Supplementary Legend. [file 41598_2022_19678_MOESM3_ESM.docx]

**Supplementary Movie 1. Freely rotatable transparent whole-brain images by CUBIC applied to Arc-dVenus Tg mice**

(A) Rotated image of Sham2 on a horizontal plane. (B) Rotatable image of UL2 in the coronal plane.
